# Supplementary material for: SRSF1-dependent inhibition of C9ORF72-repeat RNA nuclear export: genome-wide mechanisms for neuroprotection in amyotrophic lateral sclerosis
Source: Mol Neurodegener. 2021 Aug 10;16:53. doi: 10.1186/s13024-021-00475-y (PMC8353793; doi:10.1186/s13024-021-00475-y)
Supplement: Supplementary file 1 — Additional file 1. : List of qPCR primers used in this study. [file 13024_2021_475_MOESM1_ESM.docx]

**Sequences of qPCR primers used in this study**

Primers for *Drosophila Tub84b* (Ling *et al*., PLoS ONE 2011; 6:e17762):

Fwd: 5’-TGGGCCCGTCTGGACCACAA-3’

Rev: 5’-TCGCCGTCACCGGAGTCCAT-3’

Primers for *Drosophila SK* (designed using Primer-BLAST)*:*

Fwd: 5’-ACCCTGTACTGCTGTTGCC-3’

Rev: 5’-TGTACAGATTCTGATGGATGGCTT-3’

Primers for *Drosophila NAAT1* (designed using Primer-BLAST):

Fwd: 5’-CACGGGATTGGCCTTCATCT-3’

Rev: 5’-CACGGGATTGGCCTTCATCT-3’

Primers for *Drosophila DHD* (designed using Primer-BLAST):

Fwd: 5’-GTGGTCCCTGCAAGGAAATG-3’

Rev: 5’-CACCTTGTAGCGCTCCGTC-3’

Primers or Human *U1* (Hautbergue *et al*., 2017; 8:16063):

Fwd: 5’-CCATGATCACGAAGGTGGTT-3’

Rev: 5’-ATGCAGTCGAGTTTCCCACA-3’

Primers for Human *SRSF1* (Hautbergue *et al*., 2017; 8:16063):

Fwd: 5’-CCGCATCTACGTGGGTAACT-3’

Rev: 5’-TCGAACTCAACGAAGGCGAA-3

Primers for Human *C9ORF72* (Hautbergue *et al*., 2017; 8:16063):

Intron-1 Rev: 5’-GGAGAGAGGGTGGGAAAAAC-3’

Exon-3 Rev: 5’-GTCGACATGACTGCATTCCA-3’

Exon-1 For: 5’-TCAAACAGCGACAAGTTCCG-3’

Primers for Human *Usp49* (Origene):

Fwd: 5’-GGAGAATCTACGCTTGTGACCAG-3’

Rev: 5’-CGGAGAACCTGAGGTAGTCTGT-3’

Primers for Human *RSL1D1* (Origene):

Fwd: 5’-TCCGAAGACGAAATCCCACAGC-3’

Rev: 5’-GTGCTGGGATTAGGACTCTTTGC-3’

Primers for Human *MSH6* (Origene):

Fwd: 5’-AAGGACTGGCAGTCTGCTGTAG-3’

Rev: 5’-CGGCAACACAGAATTACTGGGCGA-3’

Primers for Human *RBM15* (Origene):

Fwd: 5’-CTTCCCACCTTGTGAGTTCTCC-3’

Rev: 5’-CTTCTTGTTCTCATACCTAACTCC-3’

Primers for Human *MTCL3* (Origene):

Fwd: 5’-TGCTCAAGTGCCGTCTGGAACA-3’

Rev: 5’-TGACTGTCTGCCAGGAGCTTCT-3’

Primers for Human *NUP98* (Origene):

Fwd: 5’-CCATCTATGGATGACCTGTAAA -3’

Rev: 5’- TCCGACCAATAGTGAAATCAGAGA-3’

Primers for Human *DAPK1* (Origene):

Fwd: 5’-CCAGACTGTCTTCCACCAACTC -3’

Rev: 5’- TCCTCACACTCACGTTCTCGCA-3’

Primers for Human *FN1* (Origene):

Fwd: 5’- GGACACAACGATGCTTCCTGAG-3’

Rev: 5’- ACAACACCGAGGTGACTGAGAC-3’

Primers for Human *USP19* (Origene):

Fwd: 5’-GCTGCTATCCTCAGAGTTGGCT-3’

Rev: 5’-TCATCCTCCGACTGTTGCTTCC-3’

Primers for Human *KCNN1* (Origene):

Fwd: 5’-TGCTGGTCTTCAGCATCTCCTC-3’

Rev: 5’-CGTAGCCAATGGAGAGGAAGGT-3’

Primers for Human *KCNN2* (Origene):

Fwd: 5’-GCCTTATCAGTCTCTCCACGATC-3’

Rev: 5’-CCAGTCATCTGCTCCATTGTCC-3’

Primers for Human *KCNN3* (Origene):

Fwd: 5’-GCCTTATCAGTCTGTCCACCATC-3’

Rev: 5’-TACAGGATGCGCTCGTAGGTCA-3’

Primers for Human *KCNN4* (Origene):

Fwd: 5’-CATTCCTGACCATCGGCTATGG-3’

Rev: 5’-GCCTTGTTAAACTCCAGCTTCCG-3’

Primers for Human *TRX1* (Origene):

Fwd: 5’-GTAGTTGACTTCTCAGCCACGTG-3’

Rev: 5’-CTGACAGTCATCCACATCTACTTC-3’
