# Supplementary figures and images for: SRSF1-dependent inhibition of C9ORF72-repeat RNA nuclear export: genome-wide mechanisms for neuroprotection in amyotrophic lateral sclerosis
Source: Mol Neurodegener. 2021 Aug 10;16:53. doi: 10.1186/s13024-021-00475-y (PMC8353793; doi:10.1186/s13024-021-00475-y)

**A**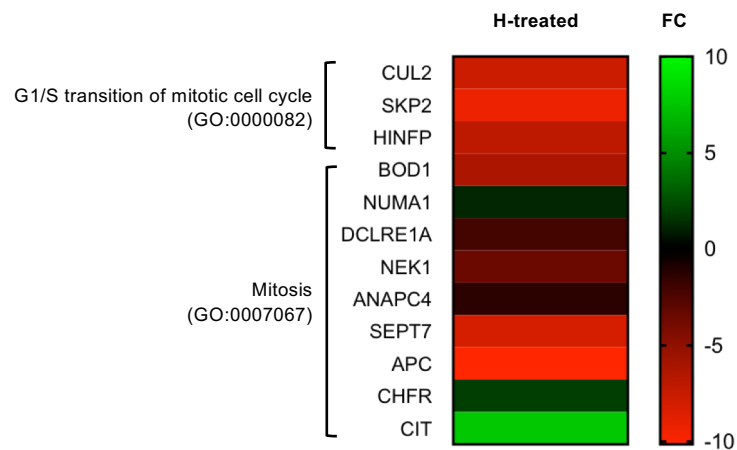**B**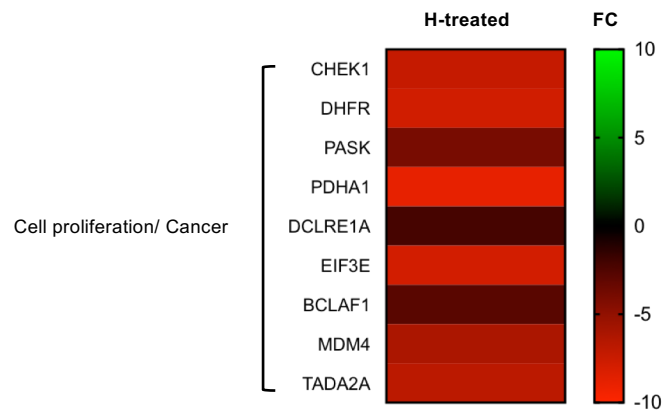

Whitfield LK et al. Nature Reviews Cancer 2006; 6:99–106  
Waldman YY et al. PLoS Genet 2013; 9(9) e1003806.

**C**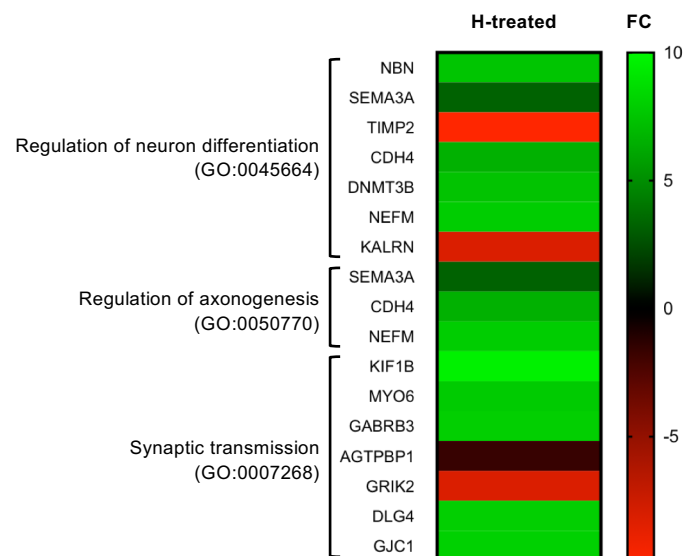

Supplement: Supplementary file 8 — Additional file 8 : Supplementary Figure 1. Downregulation of mitosis/ cell proliferation/ cancer makers and upregulation of neuronal-related functions in healthy neurons with partial depletion of SRSF1. (A) Heatmap representing the computed fold changes for the healthy-treated neurons group. Red labels down-regulated transcripts while green depicts upregulated transcripts. (B) Heatmap representing the computed fold changes for the healthy-treated neurons group. Red labels down-regulated transcripts while green depicts upregulated transcripts. (C) Heatmap representing the computed fold changes for the healthy-treated neurons group. Red labels down-regulated transcripts while green depicts upregulated transcripts. [file 13024_2021_475_MOESM8_ESM.pdf]

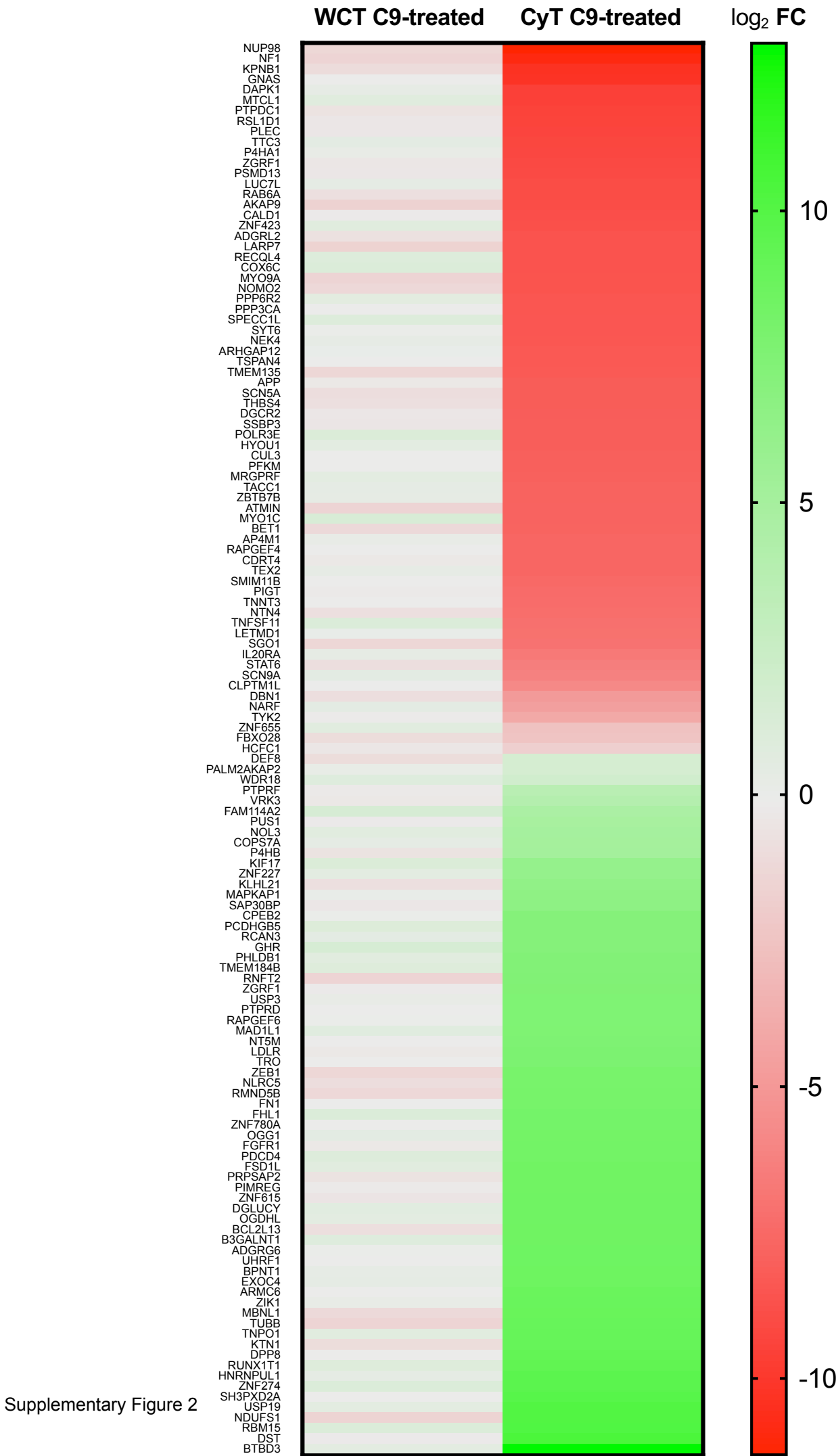

Supplementary Figure 2

Supplement: Supplementary file 12 — Additional file 12 : Supplementary Figure 2. Enlargement of Fig. 3b. mRNA nuclear export analysis upon SRSF1 depletion at mRNA and FC levels. [file 13024_2021_475_MOESM12_ESM.pdf]

Human-derived neuron  
changes conferring neuroprotection

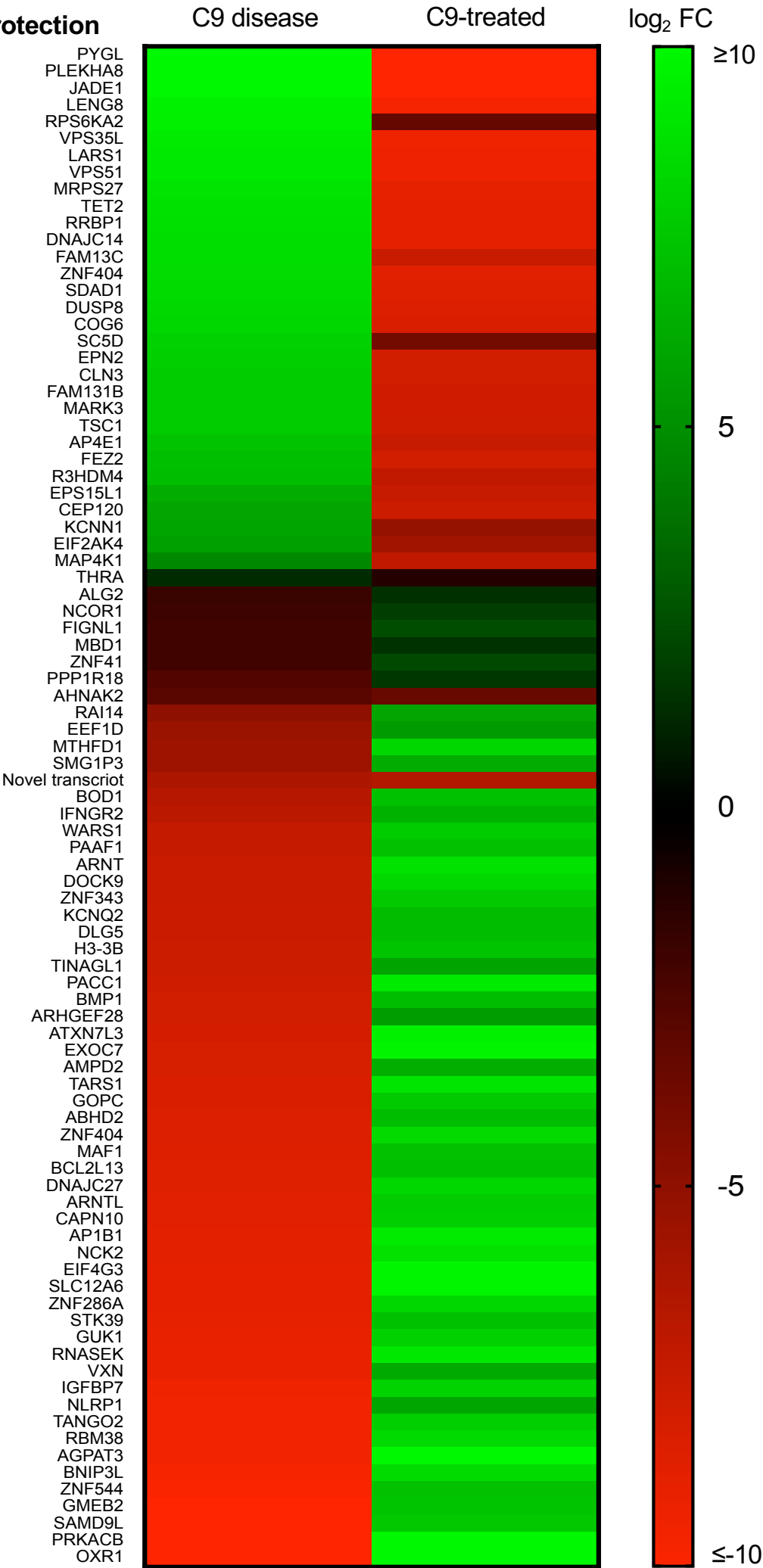

Supplementary Figure 4

Supplement: Supplementary file 16 — Additional file 16 : Supplementary Figure 4. Enlargement of Fig. 5b. C9-ALS disease-modifying signature at transcript ID and FC levels. [file 13024_2021_475_MOESM16_ESM.pdf]

**A**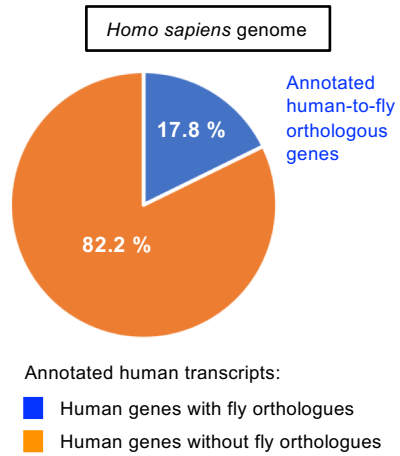**B**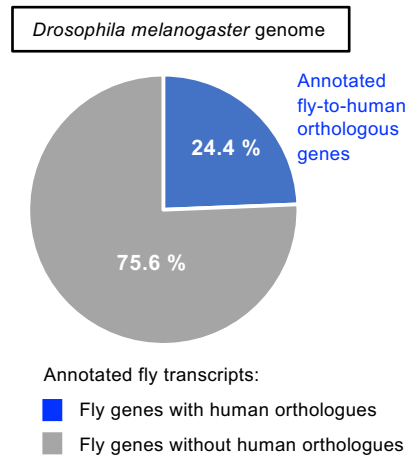

Supplement: Supplementary file 20 — Additional file 20 : Supplementary Figure 6. Orthologous genes in the human and fly genomes. BioMart (http://www.ensembl.org/biomart/martview) Ensembl Genes 99 was used with the Homo sapiens GRCh38.p13 and the Drosophila melanogaster BDGP6.28 genomes. A multispecies comparison was performed on 25.04.2020 based on (i) Homologue filters, Orthologous genes only or (ii) Homologue filters, Orthologous human Genes only. (A) 3681 human coding genes out of 20,449 have conserved fly orthologous genes. (B) 3411 fly coding genes of 13,947 have conserved human orthologous genes. List of orthologous genes are available upon request. [file 13024_2021_475_MOESM20_ESM.pdf]

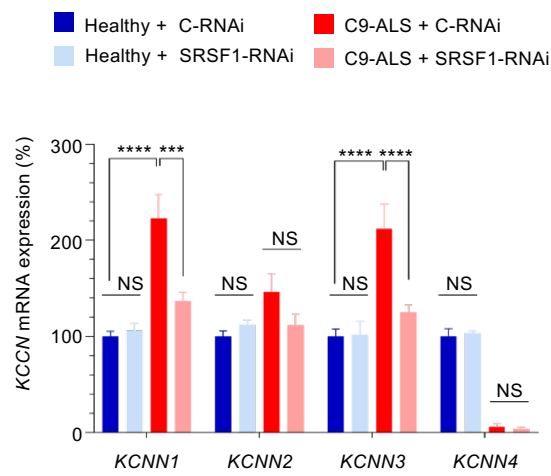

Supplement: Supplementary file 21 — Additional file 21 : Supplementary Figure 7. Quantification of KCNN1-4 mRNA expression levels in healthy and C9ORF72-ALS patient-derived neurons treated with control or SRSF1-RNAi lentivirus. Relative expression levels of KCNN1, KCNN2, KCNN3 and KCNN4 mRNAs were quantified using qRT-PCR in biological triplicates following normalization to U1 snRNA levels and to 100% for healthy neurons treated with Ctrl-RNAi (mean ± SEM; two-way ANOVA with Tukey’s correction for multiple comparisons; NS: not significant; ***: p<0.001, ****: p<0.0001; N (qRT-PCR reactions)=3). [file 13024_2021_475_MOESM21_ESM.pdf]

**A**

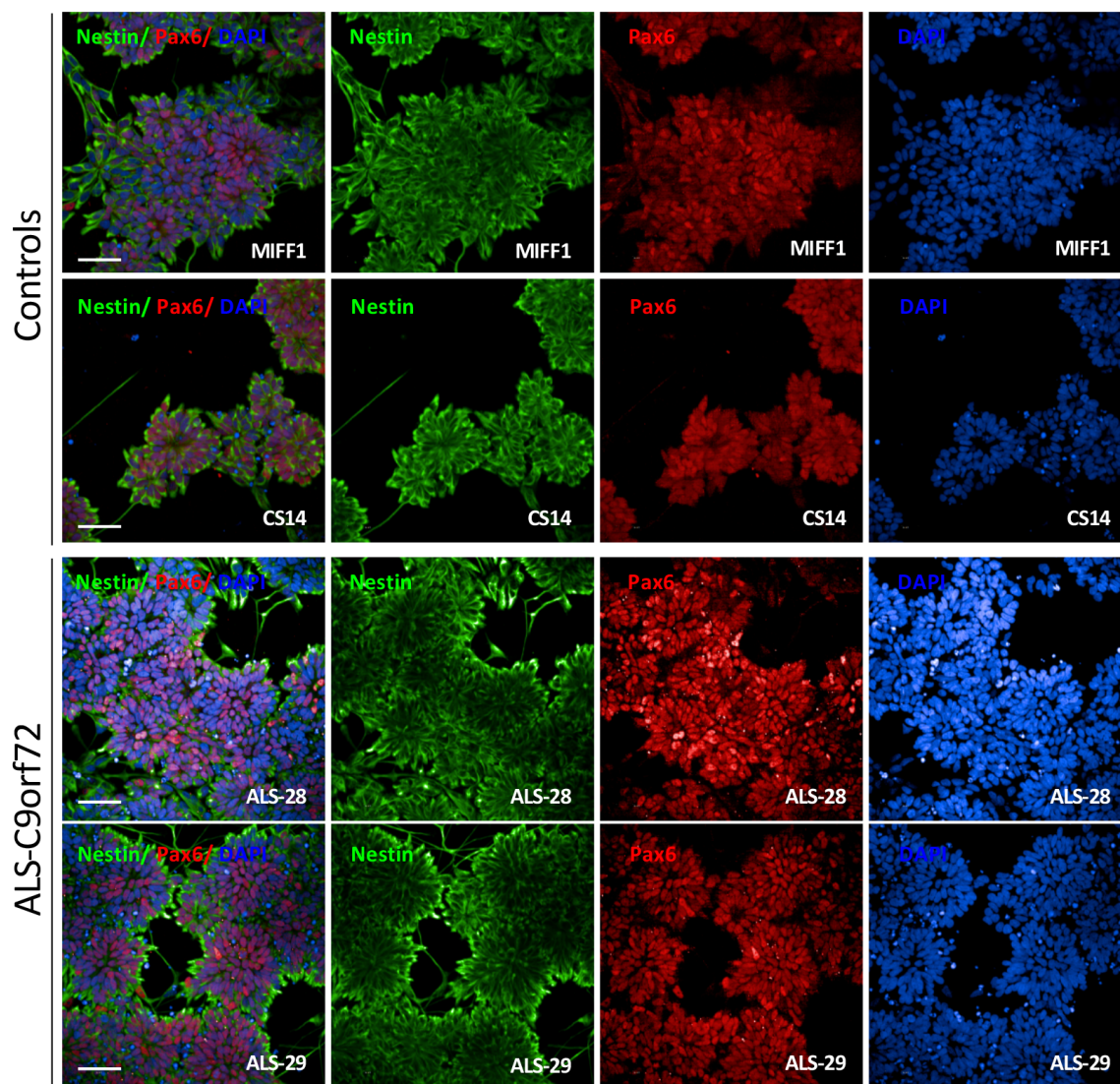

**B**

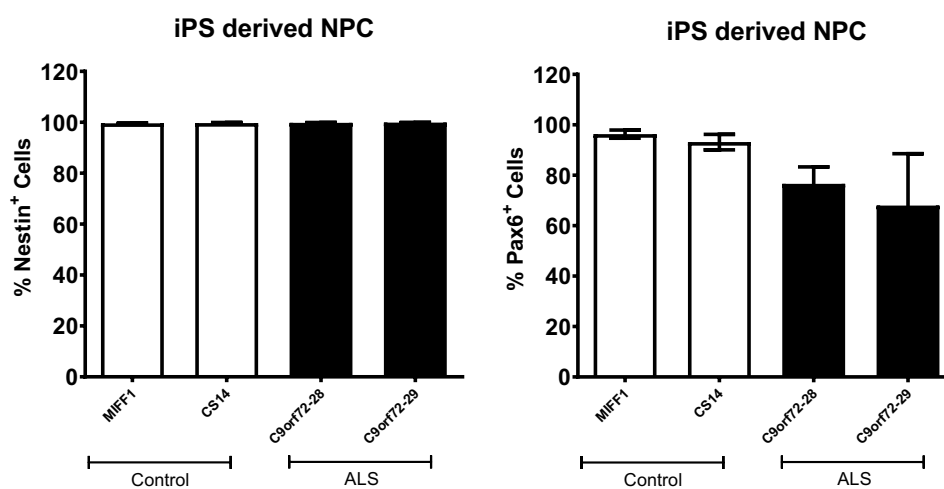

Supplement: Supplementary file 22 — Additional file 22 : Supplementary Figure 8. Characterisation of neural precursors cells (NPCs) derived from induced pluripotent stem cells (iPSCs) from healthy control and C9ORF72-ALS patients. (A) NPCs display positive staining for progenitor markers Pax6 and Nestin. Scale bars: 50 μm. (B) Quantification of cells positive for Nestin or Pax6 (n=3). [file 13024_2021_475_MOESM22_ESM.pdf]

**A**

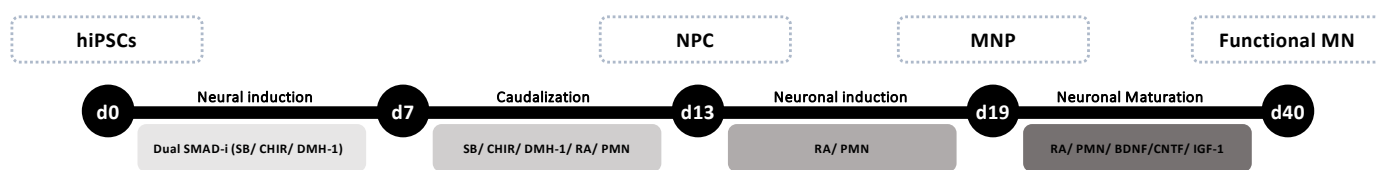

**B**

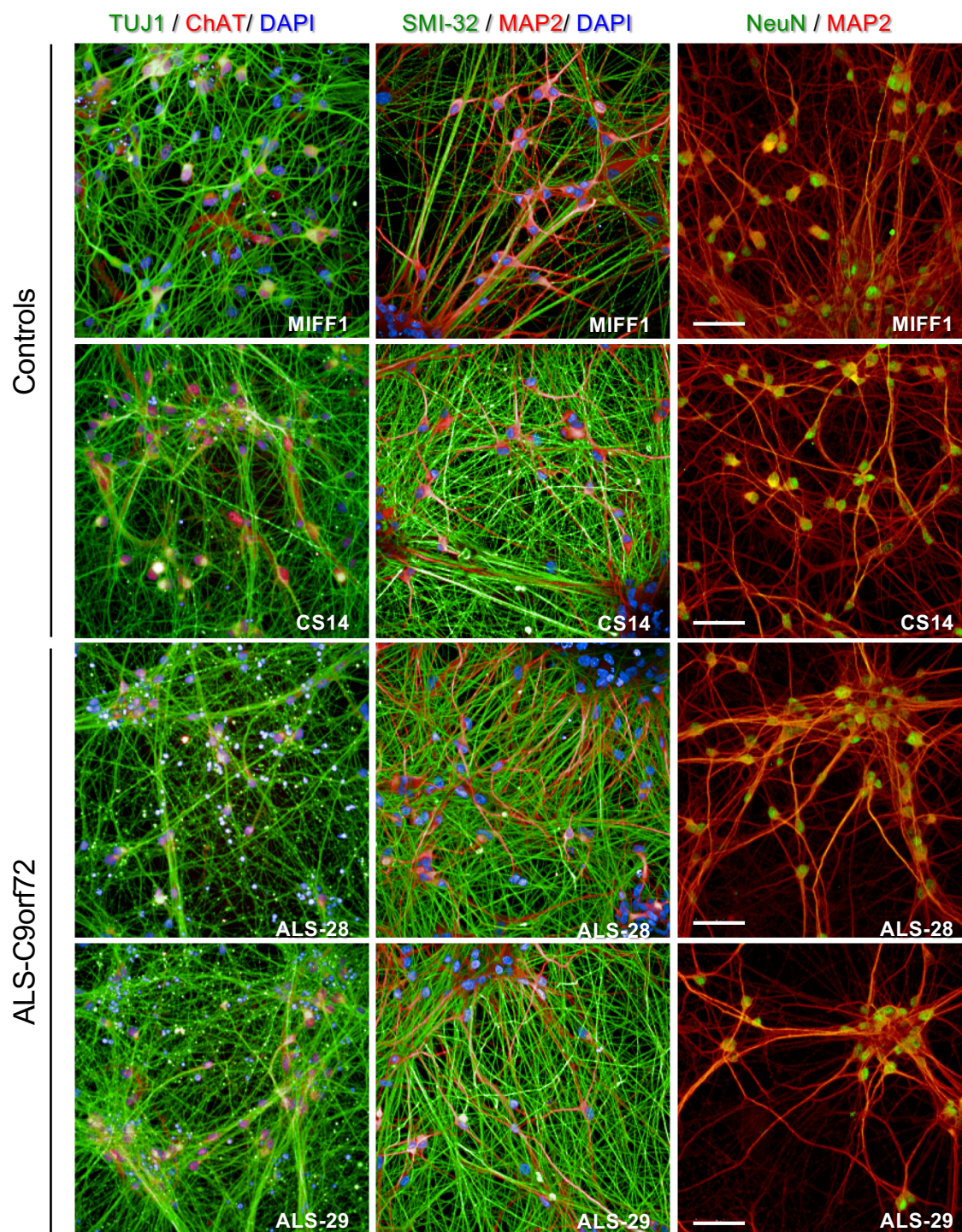

Supplement: Supplementary file 23 — Additional file 23 : Supplementary Figure 9. Differentiation of iPSCs from healthy and C9ORF72-ALS patients into functional motor neurons. (A) Schematic representation of the motor neuron differentiation protocol. (B) Representative images of motor neurons stained for the neuronal markers Tuj1, SMI-32, NeuN, MAP2 and the motor neuron specific marker ChAT. Scale bars: 50 μm. [file 13024_2021_475_MOESM23_ESM.pdf]

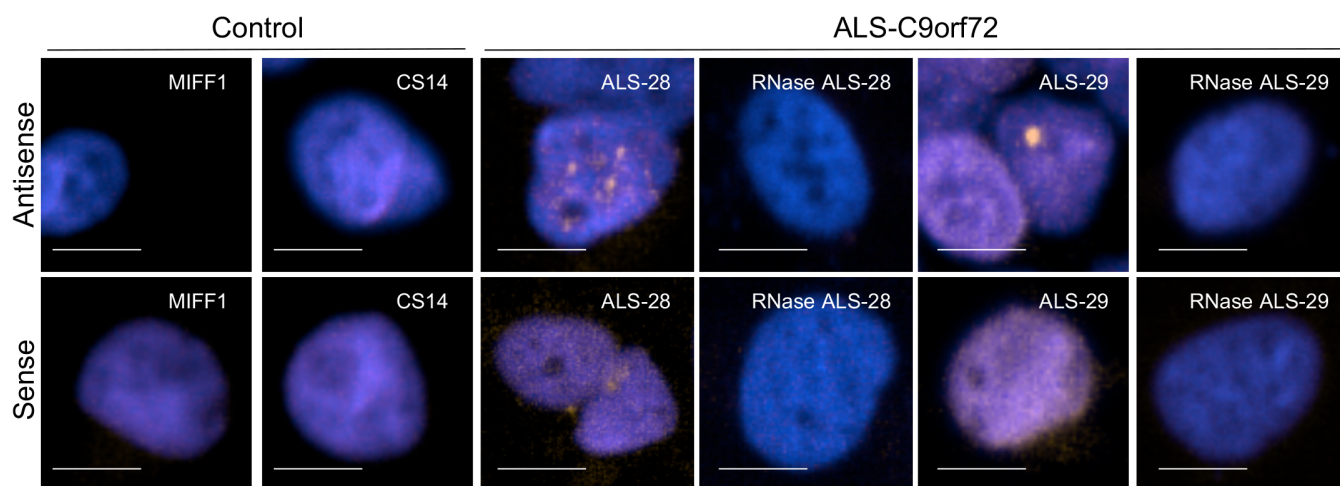

Supplement: Supplementary file 24 — Additional file 24 : Supplementary Figure 10. Motor neurons derived from C9ORF72-ALS patients develop characteristic sense and antisense RNA foci. Scale bars: 50 μm. [file 13024_2021_475_MOESM24_ESM.pdf]
