# Supplementary material for: SRSF1-dependent inhibition of C9ORF72-repeat RNA nuclear export: genome-wide mechanisms for neuroprotection in amyotrophic lateral sclerosis
Source: Mol Neurodegener. 2021 Aug 10;16:53. doi: 10.1186/s13024-021-00475-y (PMC8353793; doi:10.1186/s13024-021-00475-y)
Supplement: Supplementary file 10 — Additional file 10 : Table S8. Exon usage changes in patient-derived neurons. [file 13024_2021_475_MOESM10_ESM.docx]

**Table S8.** Statistically significant exon usage changes identified in WCT and CyT transcriptomes of human derived neurons treated with C-RNAi or SRSF1-RNAi lentivirus.

| Transcriptomes | Changes at exon level | Changes at gene level |
| --- | --- | --- |
| WCT: H_C-RNAi vs C9_C-RNAi (C9 disease) | 40 | 34 |
| WCT: C9_C-RNAi vs C9_ΔSRSF1 (C9-treated) | 0 | 0 |
| WCT: H_C-RNAi vs H_ΔSRSF1 (healthy-treated) | 68 | 61 |
|  |  |  |
| CyT: H_C-RNAi vs C9_C-RNAi (C9 disease) | 99 | 77 |
| CyT: C9_C-RNAi vs C9_ΔSRSF1 (C9-treated) | 0 | 0 |
| CyT: H_C-RNAi vs H_ΔSRSF1 (healthy-treated) | 6 | 6 |
