# Supplementary material for: SRSF1-dependent inhibition of C9ORF72-repeat RNA nuclear export: genome-wide mechanisms for neuroprotection in amyotrophic lateral sclerosis
Source: Mol Neurodegener. 2021 Aug 10;16:53. doi: 10.1186/s13024-021-00475-y (PMC8353793; doi:10.1186/s13024-021-00475-y)
Supplement: Supplementary file 19 — Additional file 19 : Supplementary Figure 5. Orthologous gene changes in the human and fly C9-disease groups. 48 orthologues show conserved direction of differential expression at gene level (DEGs) while 33 others exhibit opposite direction of changes. Green and red labels respectively correspond to up-regulation and down-regulation of the expression levels of the corresponding transcripts. [file 13024_2021_475_MOESM19_ESM.pdf]

Common orthologous gene expression changes identified in C9-disease between 1,804 human DEGs and 644 fly DEGs

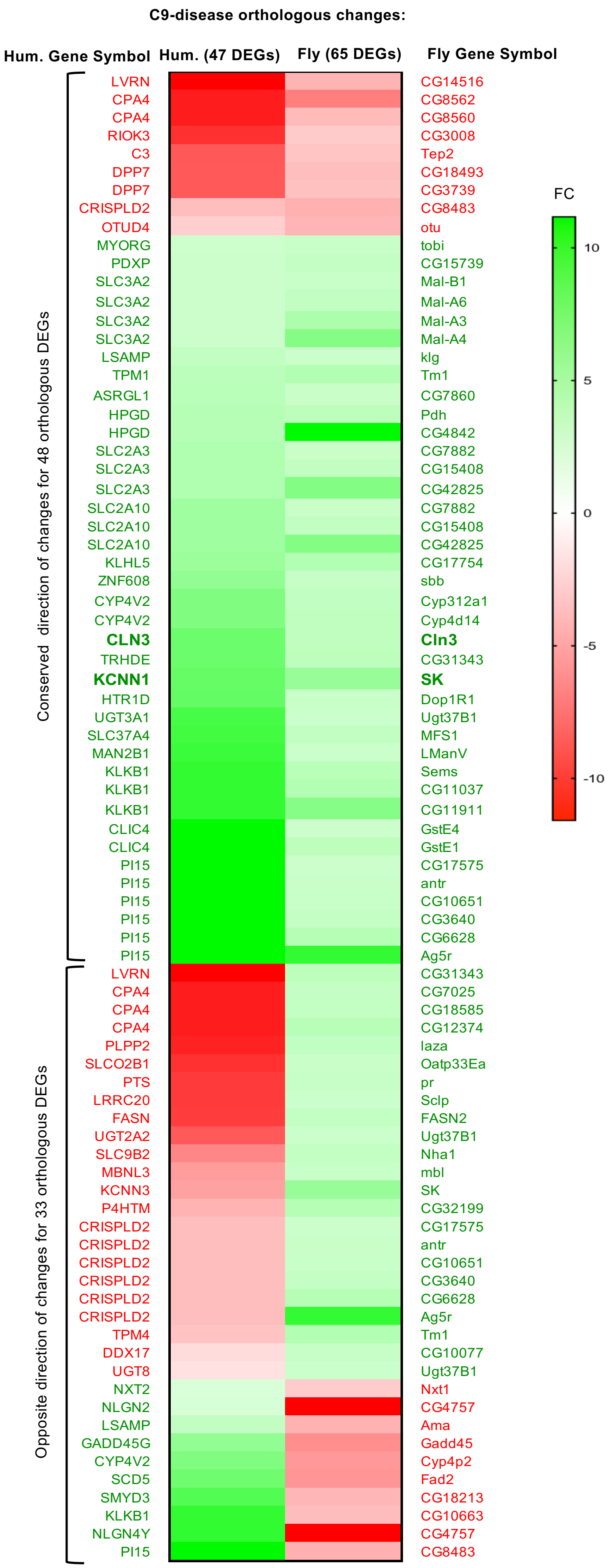

Supplementary Figure 5
