# Supplementary material for: SRSF1-dependent inhibition of C9ORF72-repeat RNA nuclear export: genome-wide mechanisms for neuroprotection in amyotrophic lateral sclerosis
Source: Mol Neurodegener. 2021 Aug 10;16:53. doi: 10.1186/s13024-021-00475-y (PMC8353793; doi:10.1186/s13024-021-00475-y)
Supplement: Supplementary file 15 — Additional file 15 : Supplementary Figure 3. Heatmap representing a clustered analysis of the C9-ALS disease-modifying transcript signature in patient-derived neurons. H: healthy lines 1, 2, 3; C9: C9ORF72-ALS lines 1, 2, 3; Δ: SRSF1 depletion. [file 13024_2021_475_MOESM15_ESM.pdf]

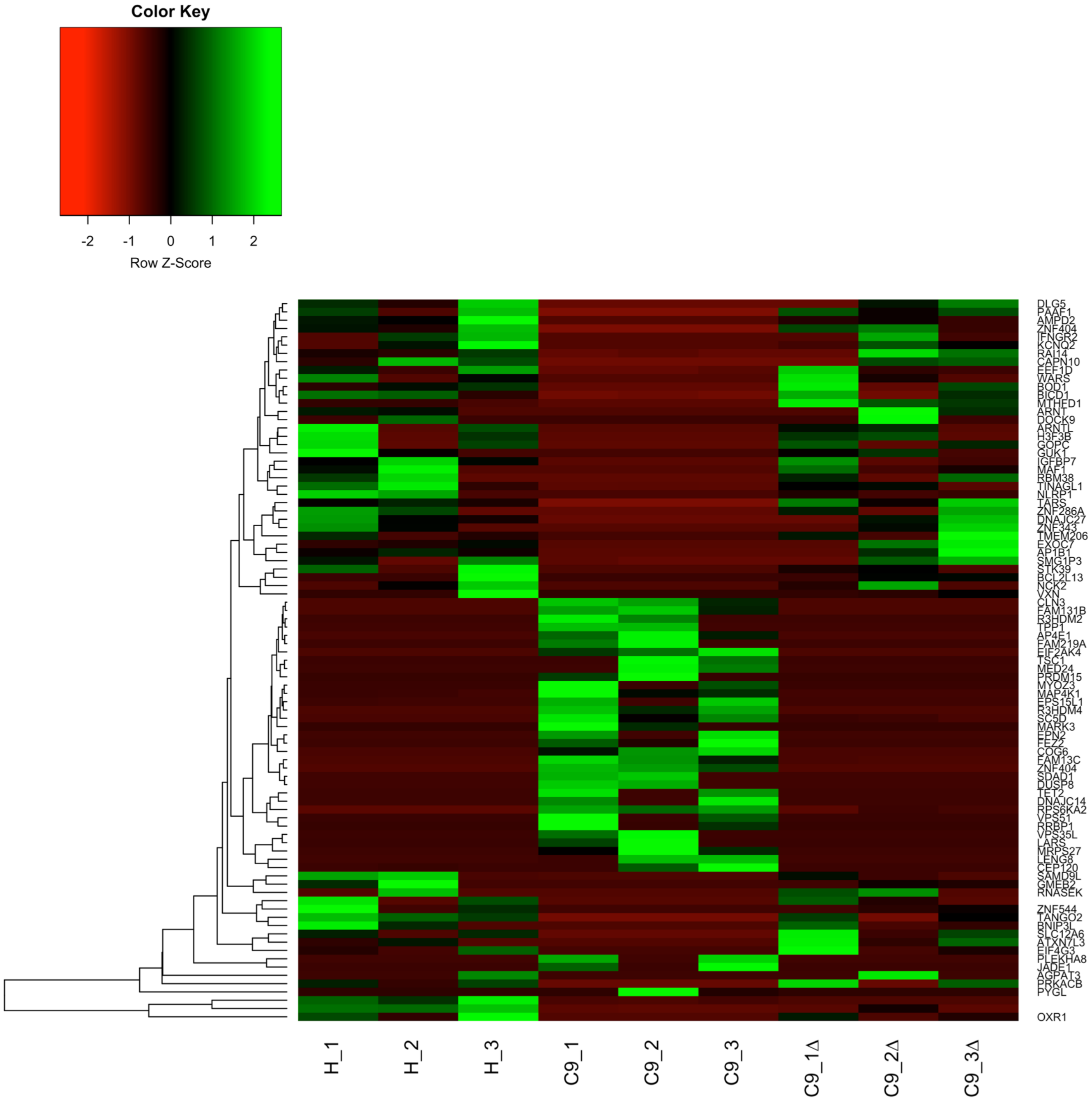

Supplementary Figure 3
